# Angiotensinogen and the Modulation of Blood Pressure

**DOI:** 10.3389/fcvm.2021.645123

**Published:** 2021-03-18

**Authors:** Zimei Shu, Jiahui Wan, Randy J. Read, Robin W. Carrell, Aiwu Zhou

**Affiliations:** ^1^Department of Pathophysiology, Shanghai Jiao Tong University School of Medicine, Shanghai, China; ^2^Department of Haematology, Cambridge Institute for Medical Research, University of Cambridge, Cambridge, United Kingdom

**Keywords:** serpin, angiotensinogen, renin, tail-in-mouth, allosteric, redox switch, hypertension, pre-eclampsia

## Abstract

The angiotensin peptides that control blood pressure are released from the non-inhibitory plasma serpin, angiotensinogen, on cleavage of its extended N-terminal tail by the specific aspartyl-protease, renin. Angiotensinogen had previously been assumed to be a passive substrate, but we describe here how recent studies reveal an inherent conformational mechanism that is critical to the cleavage and release of the angiotensin peptides and consequently to the control of blood pressure. A series of crystallographic structures of angiotensinogen and its derivative forms, together with its complexes with renin show in molecular detail how the interaction with renin triggers a profound shift of the amino-terminal tail of angiotensinogen with modulation occurring at several levels. The tail of angiotensinogen is restrained by a labile disulfide bond, with changes in its redox status affecting angiotensin release, as demonstrably so in the hypertensive complication of pregnancy, pre-eclampsia. The shift of the tail also enhances the binding of renin through a tail-in-mouth allosteric mechanism. The N-terminus is now seen to insert into a pocket equivalent to the hormone-binding site on other serpins, with helix H of angiotensinogen unwinding to form key interactions with renin. The findings explain the precise species specificity of the interaction with renin and with variant carbohydrate linkages. Overall, the studies provide new insights into the physiological regulation of angiotensin release, with an ability to respond to local tissue and temperature changes, and with the opening of strategies for the development of novel agents for the treatment of hypertension.

## Introduction

**Figure 1:**
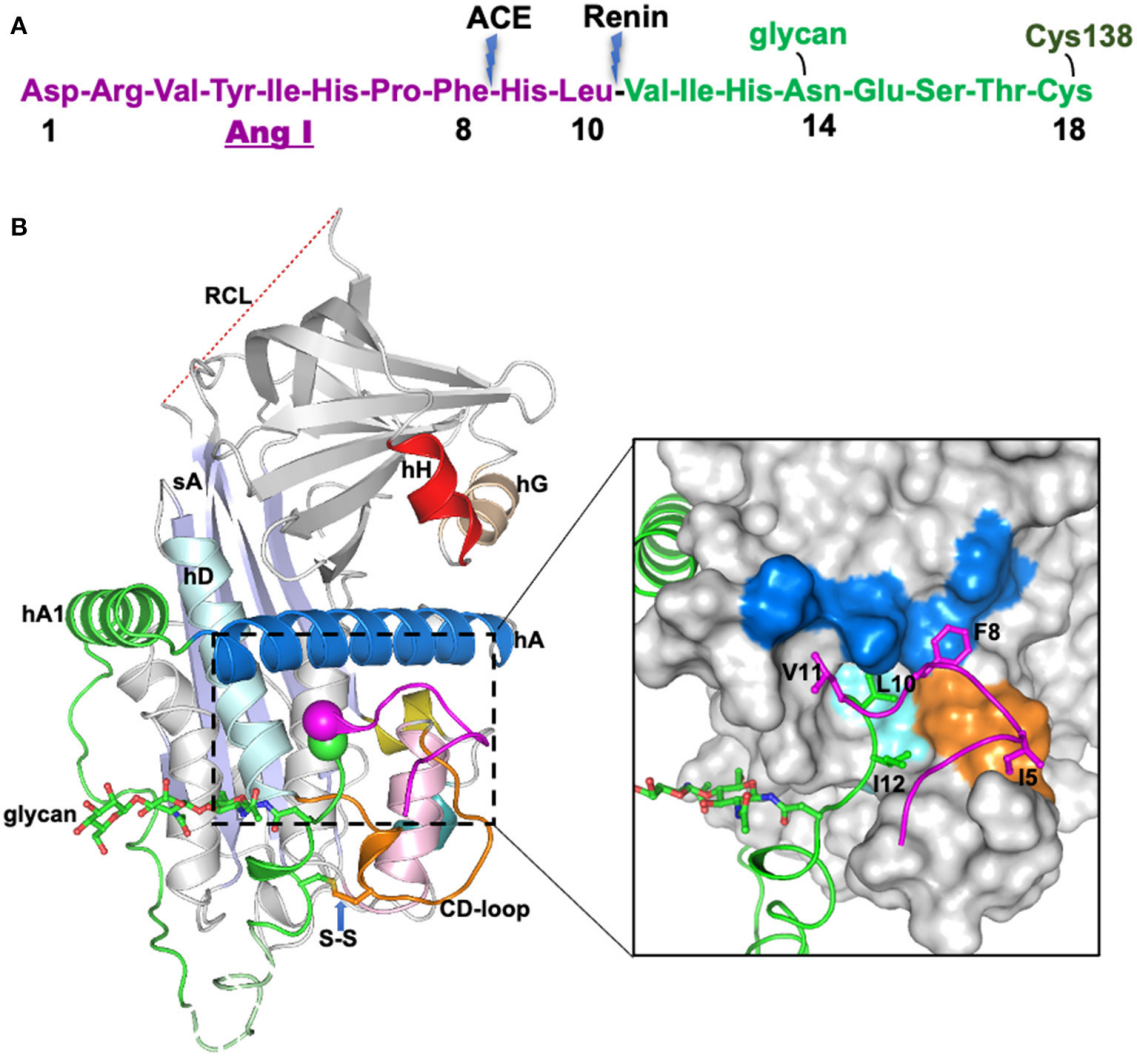
The crystal structure of human glycosylated angiotensinogen (9). **(A)** N-terminal tail sequence, indicating renin and ACE (angiotensin converting enzyme) cleavage sites, the glycosylation site and conserved disulfide bond. **(B)** Structure of angiotensinogen shown as a cartoon. Serpin template in gray, helix A in marine, A-sheet light blue, and the disordered reactive center loop (RCL) in red dashes. Angiotensin-I tail in green with the scissile bond shown as magenta and green spheres. Cys18 in the amino tail forms a disulfide bond with Cys138 of the CD-loop (brown). The glycan attached to Asn14 is shown as green sticks. The segment from Glu20-Pro29 (dashed, pale green) is disordered in the structure and modeled for illustration. Surface representation of the main body of AGT, with the extra N-terminus (1-63) shown in cartoon representation. The Angiotensin-I peptide is mainly stabilized by hydrophobic interactions with the main body (colored as yellow surface), and the scissile bond (shown as spheres) is buried in the hydrophobic cavity formed by residues in the CD-loop (V131, P132, and W133), helix A (L68, M72, L76, and F79) and helix D (L142 and V147). The figure was adapted from our previous publication (9) with modifications.

Angiotensinogen, a non-inhibitory serpin (1, 2), has a key physiological function as the carrier of the angiotensin peptides that control blood pressure. It acts in this way as a substrate, in what is the first and rate-limiting step in the renin–angiotensinogen system (RAS), with the cleavage of the N-terminal extension of angiotensinogen by the highly-specific aspartyl-protease renin. Cleavage of the N-terminus releases a decapeptide, angiotensin-I, which is then subsequently processed (Figure 1A) to give the sub-peptides that influence salt retention and vasoconstriction, and hence, control blood pressure (3, 4). Although angiotensinogen is present in the plasma in relatively high concentration (0.8 μM), its primary function is now believed to occur at a cellular level (5); with its direct role in the control of blood pressure (6) emphasized by the recent demonstration of the hypotensive response to its siRNA suppression (7).

## Structural Mechanism

The role of angiotensinogen in the RAS was for long considered as merely that of an inert substrate. The previous questioning of this passive role (6, 8), suggesting an inherently active role of angiotensinogen in the overall control of the release of angiotensin and, hence, in the modulation of blood pressure, has now been definitively confirmed by our recent structural studies (9, 10). The solving of a series of crystallographic structures at high resolution of human angiotensinogen, together with its physiologically truncated forms and complexes with renin, now provides a video view of the conformational shifts that take place on the interaction of angiotensinogen with renin. The juxtapositioning and complexing of the two molecules requires major shifts in each, not only to reveal the renin-cleavage site in angiotensinogen and position it within the active site of renin but also to give the widespread changes in each that are necessary for their highly specific interlinkage. As highlighted here, this video view of the interaction of the two molecules provides direct insights of medical and biological significance, indicating how the cleavage of angiotensinogen and release of angiotensin-I can be modulated by external factors and providing an explanation for the tight species specificity of the cleavage of angiotensinogen by renin.

## Buried Cleavage Site

The crystal structure of angiotensinogen shows that it essentially retains the typical serpin fold, including an exposed, although inert, reactive center loop. The striking difference, however, is seen in the superstructure formed by the 63-residue N-terminal extension containing the angiotensin-I decapeptide. This terminal tail is anchored to the body of angiotensinogen by extensive hydrophobic bonding including two new helices, with the renin-cleavage site at Leu10-Val11 being held in an inaccessible buried site (Figure 1B). The advantage of this buried configuration is that it protects the scissile bond in the circulating protein, with the complexity of its conformational exposure and entry into the active site cleft of renin ensuring the precise specificity of the cleavage.

## Conformational Shift in Renin Binding and Cleavage

An enlightenment from the structural findings is that the conformational shift in angiotensinogen on its interaction with renin is seen to not only expose the angiotensin cleavage site but also to involve widespread changes that allow the complementary binding of the two molecules. This tight and extensive interlinkage of renin and angiotensinogen ensures the precise entry of the scissile bond into the active site cleft of renin and explains the proteolytic specificity of the release mechanism. Major conformational shifts take place on the docking of renin, with the angiotensin segment of the N-terminal tail of angiotensinogen being competitively displaced by 10–20 Å from its linkages to the body of the molecule. This is accompanied by a 10-Å displacement of the CD loop of angiotensinogen, which would otherwise sterically block the binding of renin, the two concerted movements being linked by a conserved disulfide bond. The widespread nature of other changes that take place on the binding of the two molecules has been further revealed in the most recent high-resolution structures (10), which show an accompanying rearrangement of helix H of angiotensinogen to allow more extensive bonding between the two molecules.

## Selectivity of the Release Mechanism

The requirement of widespread bonding explains the highly specific interaction of angiotensinogen and renin and emphasizes the tight control exercised over the release of angiotensin. Evidence of this selectivity of release comes from the observed difference in the kinetics of the release of the angiotensin decapeptide from synthetic peptides (11) or from a surrogate serpin carrier (12), as compared to the release from angiotensinogen. This is even more evident in the interspecies selectivity of the interaction with renin, thus human renin will only cleave human angiotensinogen and not that of the mouse or rat (13). The critical factor in this selectivity has now been shown (10) to be due to changes not as expected from other protease studies in the residues surrounding the scissile site, but rather in more peripheral residues involved in the body-to-body interface between the two molecules.

## Modulation: Oxidation and Pre-Eclampsia

The structural findings as well as showing the precision of the cleavage of angiotensinogen by renin also indicated the likelihood that the conformational changes involved could in themselves readily allow a modulation of angiotensin release. With this in mind, attention focused on the disulfide bridge that links the movement of the N-terminus of angiotensinogen and the accompanying shift of the CD loop necessary for the body-to-body binding of renin (9). This conserved S-S bridge, between Cys18 in the N-terminus to Cys138 in the body of angiotensinogen, was known from earlier biochemical studies to have a critical functional role (12), and to be subject to external oxidation (14). Moreover, there were recurrent references in the literature to the occurrence of hypertensive crises at times of oxidative stress (15, 16). Could the oxidation of the disulfide bridge affect the cleavage by renin and the release of angiotensin?

Support for this came with the demonstration (9) that this linking-disulfide existed in humans in a balanced equilibrium between its oxidized (bridged) and reduced (unbridged) states. Blood plasma samples from healthy individuals, regardless of gender or age, showed a remarkably consistent reduced-to-oxidized ratio for the S-S bridge, near 40:60, with the redox poise readily allowing a switch between the two forms. Significantly the switch from the reduced to the oxidized form results in a 4-fold increase in the catalytic release of angiotensin (9). Taken together, these findings strongly imply a modulatory mechanism with the deduction being that episodic hypertension could be triggered by the oxidative conversion of angiotensinogen to its more active bridged form. The investigation of this proposal using plasma assays was challenging, as the oxidative switch predictably occurs diffusely, in renal and vascular tissues, rather than in the circulation (5). Confirmation came however, from the more focal oxidative stress that occurs in the placenta and underlies the hypertensive crises of pregnancy: pre-eclampsia. Here, evident changes were demonstrable in maternal plasma samples from pre-eclamptic pregnancies, with a clear increase in the oxidized form as compared to carefully matched normal-pregnancy controls (9). The results in this initial study have now been convincingly supported by subsequent quantitative assays in pre-eclamptics, showing a consistent increase in the proportion of oxidized angiotensinogen (17, 18) coupled with a fall in plasma antioxidants (19). The 4-fold increase in the catalytic efficiency of release of angiotensin by renin from oxidized angiotensinogen may seem small, but evidence that it is a sufficient cause of the resultant hypertension comes from the previous finding of a similar but even smaller increase in activity associated with hypertension, in a family with an angiotensinogen cleavage-site mutation and a history of pre-eclampsia (20).

These findings clearly establish the contribution of redox changes to the regulation of blood pressure, but oxidation is just one factor in the regulation of angiotensin release from angiotensinogen. The less active unbridged form of angiotensinogen, with reduced sulfydryls, is also demonstrably stabilized by nitrosylation (9) in keeping with the known hypotensive action of nitric oxide. Other adjustments of the efficiency of the renin-release of angiotensin were shown (10) to arise from variations of glycan composition, notably so at Asn14 in close proximity to the renin-cleavage site at Leu10. The overall clinical and physiological message is that angiotensinogen is not an inert substrate. It inherently contains complex and responsive adaptations that make it a key initiator in the regulation of blood pressure.

## Why a Serpin?

The bonus from the more recent structures of the complex of renin with angiotensinogen (10) is the answer they provide to what has long been an intriguing puzzle. Why has evolution selected the complex serpin framework as the carrier of the angiotensin decapeptide?

The serpins are an ancient protein superfamily, the members of which have evolved over millions of years from their origins as protease inhibitors in early unicellular organisms (21). The survival and now predominance of the serpins in all forms of life is dependent on the efficiency of their inhibitory mechanism, which irreversibly entraps a target protease. The entrapment mechanism involves a profound conformational change, with a large 70-Å movement of the cleaved reactive loop to incorporate it into the main beta sheet of the molecule (22). This transition, from a metastable stressed (S) form to a hyperstable relaxed (R) form, has been conserved in most but not all the later members of the family. In particular, three of the principal serpins in human plasma are carriers of essential hormones: the thyroxine and corticosteroid binding globulins, TBG and CBG, and angiotensinogen. All three carrier serpins have lost their functions as protease inhibitors although TBG and CBG have retained the ability to undergo the S-to-R transition (23). The conformational shift that accompanies the movement of the reactive center loop in TBG and CBG is seen to be directly transmitted to the hormone-binding site in each and, hence, to affect the binding and release of the hormones. Angiotensinogen does not, however, undergo the S-to-R transition in stability (2), but nevertheless, it was confidently expected that, as with TBG and CBG, the release of its hormone would be modulated by the inherent serpin mechanism. Repeated structures of reactive-center cleaved and other forms of angiotensinogen failed however to show any of the conformational shifts expected from the movement of the cleaved loop into the body of the molecule. This was a puzzling disappointment. Based on a primordial form present in lampreys (24), angiotensinogen is believed to have originated as an add-on adaptation of an active protease-inhibitory serpin, so there had been good reason to expect that, as with TBG and CBG, the justification for this adaptation would be an accompanying regulatory advantage.

## Tail-in-mouth Modulatory Mechanism and the Release of Angiotensin

**Figure 2:**
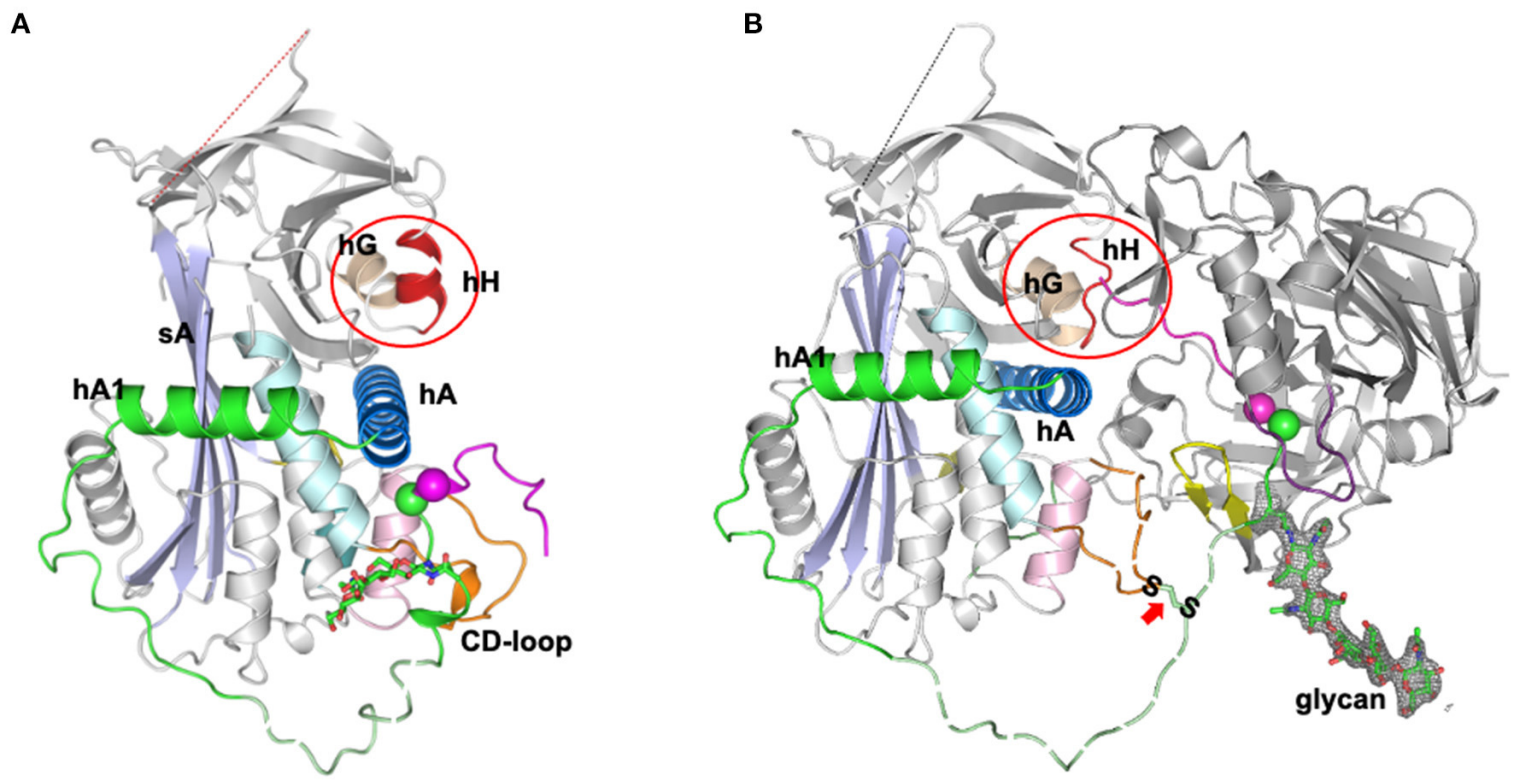
Tail-into-mouth shift of N-terminal tail on binding with Renin (10). **(A)** Side view of angiotensinogen showing the rearrangement of the tail (magenta and green) that takes place on the complexing with renin in **(B)**. The scissile bond, Leu10-Phe11 (magenta and green spheres) becomes exposed and the tail is anchored at its terminus by docking into what in other serpins is the hormone-binding pocket, between helix H, the B beta-sheet, and helix A (as circled). Docking of the terminus is accompanied by an unfolding of helix H to reveal sidechains essentially involved in the binding of renin. The S-S bridge between Cys18 of the tail and Cys138 of the displaced CD loop of angiotensinogen is arrowed. Also apparent: the potential influence on cleavage kinetics of glycan variations at Asn14, adjacent to the scissile bond. The clear electron density for the glycan linked to Asn14 is shown in gray mesh and the fragments without clear electron density are shown with dashed lines for illustration. The figure was adapted from our previous publication (10) with modifications.

An unexpected answer came from the later high-resolution structures of the complex of angiotensinogen and renin (10), which showed how angiotensinogen had indeed adapted a subtle feature of the serpin mechanism to allow a fine-tuning of the release of the angiotensin peptide. The first few residues of the amino-tail of angiotensinogen were seen in the complex to extend beyond the active cleft of renin and to insert into the equivalent in angiotensinogen of what in TBG and CBG is the hormone-binding pocket (Figure 2). This “tail-in-mouth” action in angiotensinogen requires an unfolding of helix H, which forms one wall of the binding pocket, with the unfolding revealing key sites involved in the bonding that links the bodies of the renin and angiotensinogen. In this way the interaction between the two molecules can be seen to be dependent on widespread conformational changes that allow the complementary binding of the two, with integral to this the movement of the scissile bond of angiotensinogen into the active site of renin and the insertion of the initial residues of the N-terminus into the “helix H” pocket of angiotensinogen. This latter, newly-recognized aspect of the interaction with renin is of critical regulatory significance. The efficiency of cleavage and release of the angiotensin decapeptide, a rate-limiting step in the control of blood pressure, is now seen to be dependent on the completion of the renin-angiotensinogen interface, revealed by the unfolding of the H helix. Conversely the cleavage of the angiotensin decapeptide from the tail will cause a reversion of this complementarity, with an accompanying dissociation of the renin from angiotensinogen and the release of angiotensin-I. Confirmation of the mechanism of this dissociation comes from the observed loss of renin-binding affinity in post-cleavage (des-angiotensin) angiotensinogen (10).

## Temperature Sensitivity of Angiotensin Release?

The modulatory potential of this tail-in-mouth mechanism is clear. Any competitive blockage of the serpin “hormone”-pocket of angiotensinogen or decrease in its binding-affinity will hinder the interaction with renin, with a predictable decrease in angiotensin release and, hence, hypotensive consequences. A direct implication follows from recent studies with the thyroxine and corticosteroid binding globulins, TBG and CBG (25–28). These hormone-carrying serpins show how the affinity of the hormone binding-pocket is inherently responsive to changes in temperature, even in their conformationally inactive forms. The temperature sensitivity is much more so however in active TBG and CBG, with the small equilibrated movements of the reactive center loop nudging into and out of the main beta sheet of the molecule directly affecting the flexibility of the binding-pocket. These coupled movements provide a clinically demonstrable “molecular thermocouple” (29, 30), accelerated as temperatures rise above 37°C to give with TBG and CBG a markedly increased release of thyroxine and cortisol in fevers.

The control of blood pressure is multifactorial but the retention in angiotensinogen of this thermally-responsive flexibility of the binding-pocket is likely to contribute to the immediacy of fluctuations in blood pressure observed with variations in temperature. Increased body temperatures, with a lowering of binding affinity, will predictably hinder the interaction with renin and, hence, contribute to a decreased release of angiotensin, in keeping with the vasodilation and decreases in blood pressure that occur in fevers (31). Such temperature-sensitive changes in affinity are similarly compatible with other fluctuations in blood pressure with ambient temperatures (32); conversely so with a predictable increased affinity at lower temperatures, in keeping with the observed prompt rise in central aortic blood pressure after even short-term exposure to winter cold (33).

## Conclusions

The new structural understandings of the mechanism of cleavage and release of angiotensin from angiotensinogen have profound medical and physiological implications. Angiotensinogen has long been known as the ultimate source of angiotensin but what is now revealed by the structures of its complexes with renin is angiotensinogen's direct role in regulating the cleavage and release of the peptide and, hence, in the control of blood pressure. This inherent ability to modulate function in response to local tissue changes, as is also so with the plasma carriers of thyroxine and corticosteroids (34), explains why the conformationally flexible serpin framework has been selected as the carrier of angiotensin. The conformational shifts required for the release of angiotensin-I involve not just the exposure of the buried renin-cleavage site but also an accompanying rearrangement of the wider sites required for the body-to-body interface of renin with angiotensinogen. In particular, optimal kinetics for the cleavage and release depends on the precise repositioning of the renin-cleavage bond at Leu10-Val11. This is held in its exposed configuration anchored between the S-S bridge at Cys18 and the N-terminus of the tail bound to the helix H pocket (Figure 2).

The realization that each of these anchors can be readily modified by external influences emphasizes the modulatory role of angiotensinogen and opens new prospects for the investigation of the causes and ultimately the treatment of hypertension. The S-S bridge is demonstrably labile *in vivo* and is readily opened and re-formed—reduced and oxidized—by local redox fluctuations. This notably occurs with the placental oxidative stress and consequent hypertension in pre-eclampsia (9). The challenge now is to demonstrate whether such changes, occurring at a wider tissue level, are a contributory cause of hypertension in general. Blocking of the more active oxidized form of angiotensinogen has been shown to occur with nitrosylation—to what extent does this explain the hypotensive action of nitric oxide? More questions follow from the recognition of the tail-into-mouth mechanism that establishes the other anchor of the cleavage site. The vestigial conformational mechanism involved, with associated changes in the affinity of the binding-pocket, has been well studied (27) in other ligand-binding serpins, in antithrombin as well as TBG and CBG. Is the binding of the tail in angiotensinogen similarly sensitive to small changes in temperature? Is the hypotension observed to occur with hypothermia due to a decrease in the affinity of the binding-pocket, and hence in a decrease in angiotensin release? Predictably the binding site in angiotensinogen, as with the other serpins, will be subject to competitive blocking, by drugs and other small molecules, and to modulation by its interaction with tissue receptors.

What can be concluded with confidence is that the recognition of the structurally well-defined helix H binding-pocket in angiotensinogen now provides a basis for the design of new agents to attenuate angiotensin release and thus alleviate hypertension.

## Author Contributions

This paper was conceived and written by RWC, RJR, and AZ with input from ZS and JW. ZS and JW prepared illustrations for the paper. All authors contributed to the article and approved the submitted version.

## Conflict of Interest

The authors declare that the research was conducted in the absence of any commercial or financial relationships that could be construed as a potential conflict of interest.
